# Sustained CO_2_-photoreduction activity and high selectivity over Mn, C-codoped ZnO core-triple shell hollow spheres

**DOI:** 10.1038/s41467-021-25007-6

**Published:** 2021-08-16

**Authors:** Mahmoud Sayed, Feiyan Xu, Panyong Kuang, Jingxiang Low, Shengyao Wang, Liuyang Zhang, Jiaguo Yu

**Affiliations:** 1 State Key Laboratory of Advanced Technology for Materials Synthesis and Processing, Wuhan University of Technology, Wuhan, 430070, P. R. China; 2 Laboratory of Solar Fuel, Faculty of Materials Science and Chemistry, China University of Geosciences, Wuhan, 430074, P. R. China; 3 Chemistry department, Faculty of Science, Fayoum University, Fayoum, 63514, Egypt; 4 College of Science, Huazhong Agricultural University, Wuhan, 430070, P. R. China

**Keywords:** Heterogeneous catalysis, Photocatalysis, Photocatalysis

## Abstract

Solar conversion of CO_2_ into energy-rich products is one of the sustainable solutions to lessen the global energy shortage and environmental crisis. Pitifully, it is still challenging to attain reliable and affordable CO_2_ conversion. Herein, we demonstrate a facile one-pot approach to design core-triple shell Mn, C-codoped ZnO hollow spheres as efficient photocatalysts for CO_2_ reduction. The Mn ions, with switchable valence states, function as “ionized cocatalyst” to promote the CO_2_ adsorption and light harvesting of the system. Besides, they can capture photogenerated electrons from the conduction band of ZnO and provide the electrons for CO_2_ reduction. This process is continuous due to the switchable valence states of Mn ions. Benefiting from such unique features, the prepared photocatalysts demonstrated fairly good CO_2_ conversion performance. This work is endeavoured to shed light on the role of ionized cocatalyst towards sustainable energy production.

## Abstract (web summary)

Photoreduction of CO_2_ into energy-rich products is a sustainable solution to lessen the global energy and environmental crisis. Here the authors show that Mn ions in Mn, C-codoped ZnO hollow spheres function as “ionized cocatalyst” to promote the CO_2_ adsorption and light harvesting to boost the CO_2_ photoreduction activity.

## Introduction

Worldwide consumption of fossil fuels is unambiguously accountable for two critical issues, *i.e.* global warming by the augmented greenhouse gases (especially CO_2_) emission and depletion of such non-renewable sources^1–6^. Nowadays, it becomes a global appeal to develop sustainable energy grids based on renewable energy sources such as solar, wind, and geothermal energies^7,8^. Reliable conversion of CO_2_ by the aid of solar light to energy-rich fuels (*i.e.* artificial photosynthesis) is an ideal solution for simultaneously relieving both energy and environmental crises^9–11^. Albeit great efforts have been devoted to exploring the potential of artificial photosynthetic materials for CO_2_ conversion, its efficiency is still unsatisfactory due to difficulties in activating thermodynamically stable CO_2_^12–14^. Therefore, it is critical to construct suitable photocatalysts and coupled with active cocatalysts. Cocatalysts can effectively promote the separation and transfer of photogenerated charge carriers to attain appealing CO_2_ reduction ability^11,15,16^.

Transition metal-based materials, such as ions, oxides, sulfides, and phosphides, exhibited outstanding potentials as cocatalysts for both oxidation and reduction reactions^17–21^. They can not only effectively lower the barrier for CO_2_ activation but also promote the separation of photogenerated charge carriers^22,23^. To date, most cocatalysts are in solid state. We wonder whether ionized cocatalysts which are embedded in the lattice of photocatalyst can also serve as a cocatalyst. They can become active sites for the photocatalytic CO_2_ reduction. Due to their multiple oxidation states, they can swap electrons with photocatalyst as well as CO_2_. Besides, the introduction of ionized cocatalyst avoids the difficulty of loading a cocatlyst on the photocatalyst. And the difficulty in maintaining intimate contact no longer exists.

Intricate hollow structures with tailored compositions have surpassed their solid counterparts in widespread applications. However, the fabrication of such intricate structures is still an arduous challenge. Multishell hollow structures (MSHSs) are characterized by manifold features such as large specific surface area, low density, as well as reduced mass and charge diffusion lengths^24^. All of these endow MSHSs with potential in multiple applications, such as photocatalysis, energy storage and conversion, drug delivery and sensors^25–27^. Concerning CO_2_ photoreduction (PR), MSHSs possess prodigious advantages over their solid counterparts. These advantages particularly spring from esthetic shell layers. For one thing, their large surface area promotes CO_2_ adsorption and surface redox reactions on both sides of the shells. For another, they provide a reduced path length for charge carriers, favoring charge migration and separation^28,29^. All these merits make MSHSs ideal support materials for anchoring photocatalysts aiming at designing elaborate catalytic system for CO_2_ PR. However, the fabrication of such MSHSs is still an arduous challenge.

**Fig. 1:**
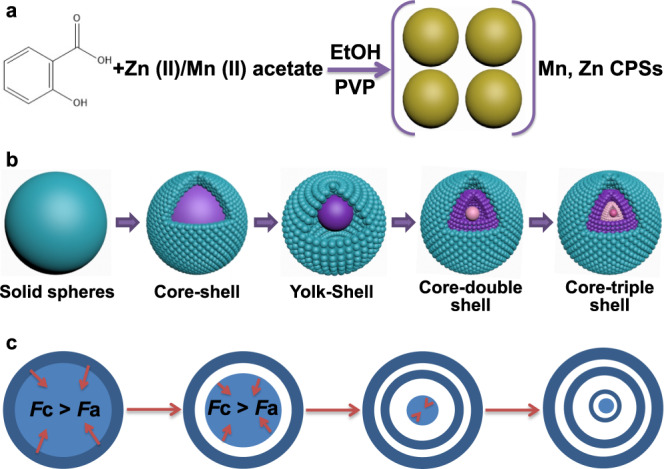
Schematic illustration of Mn, C-ZnO CTHSs formation process. **a** Preparation of Mn, Zn-CPSs from solvothermal treatment of zinc acetate and manganese acetate with salicylic acid in the presence of PVP. **b** Step-by-step conversion of Mn, Zn-CPSs into Mn, C-codoped ZnO CTSHSs upon calcination at 550 °C for 3 h. **c** The underlying driving force for such hollow structure formation.

Herein, we develop a facile one-pot coordination polymer (CP) strategy to fabricate Mn, C-codoped ZnO core–triple shell hollow spheres (Mn, C-ZnO CTSHSs). The process involves two steps: (i) formulation of Zn, Mn CP via interaction of salicylate ligand with the corresponding metal ions in the presence of polyvinylpyrrolidone (PVP) and (ii) air calcination of the resultant Zn, Mn CP (Fig. 1). The obtained photocatalyst with fascinating core–multishell morphology and tailored composition affords adequate CO_2_ reduction activity, *i.e.* two times higher than commercial ZnO. Such enhanced activity is ascribed to the improved light absorption, favorable transfer of mass and charges across the thin shell layers, and aided CO_2_ adsorption and activation. Interestingly, Mn species formed in the system as substituent of Zn ions in the ZnO lattice play an indispensable role during CO_2_ PR. They act as active centers for CO_2_ adsorption and subsequent favored CO_2_ reduction by suppressing hydrogen evolution. Most importantly, in situ X-ray photoelectron spectroscopy results reveal that Mn species can restore their primal oxidation state after CO_2_ PR without external treatment or subsidiary reducing agents. Instead, Mn^(*n*+1)+^ species can extract one photogenerated electron from the system to regenerate the original Mn^*n*+^ moieties. The switching between Mn^(*n*+1)+^/Mn^*n*+^ is recognizable by virtue of the multiple oxidation states of Mn and affordability of photogenerated electrons. These results suggest that ionized Mn can act as a durable and efficient ionized cocatalyst toward practical CO_2_ PR.

## Results and discussions

### Formulation and characterization of Mn, C-ZnO CTSHSs

The core–triple shell hollow spheres are obtained *via* a facile one-pot solvothermal preparation of Mn, Zn CP spheres (Mn, Zn-CPSs) followed by controlled air-annealing as depicted in Fig. 1. The CPSs as precursors were firstly formulated by the coordination interaction between Zn- and Mn-acetate and salicylate ligands (Fig. 1a). The carboxylate groups of salicylic acid play a significant role that they can coordinate to metal ions, enabling a delicate control over the composition. Moreover, this method is universal to most of the transition metals because non-selectivity is witnessed for salicylic acid^30^.

Fourier transform infrared spectroscopy (FTIR) spectra of the Mn, Zn-CPSs (Supplementary Fig. 1) evidenced the interaction between deprotonated carboxylate groups of salicylate ligands and metal ions. Such interaction is assured after distinctive intensity drop of the corresponding C=O peak centered at 1657 cm^–1^ and a slight shift to 1654 cm^–1^^30^. The deprotonation of the carboxylate groups is very critical to boost such coordination interaction. As a strong conjugated base, acetate anions can deprotonate the carboxylate groups of salicylic acid and thus enable reliable coordination between salicylate ligand and metal cations. To confirm this assumption, we replaced Zn- and Mn-acetates by their sulfates, nitrates, or chlorides and followed the same preparation recipe. All of them fail to fabricate such spherical architecture. We attribute such failure to the inability of the corresponding anions to deprotonate carboxylate groups of salicylic acid. After formation of Mn, Zn-CPSs, the generation of hollow structures derives from the calcination process. In this step, the Mn, Zn-CPS precursor is subjected to air calcination at 550 °C, a step that culminates the unique CTSHS morphology by heterogeneous contraction process (Fig. 1b, c).

**Fig. 2:**
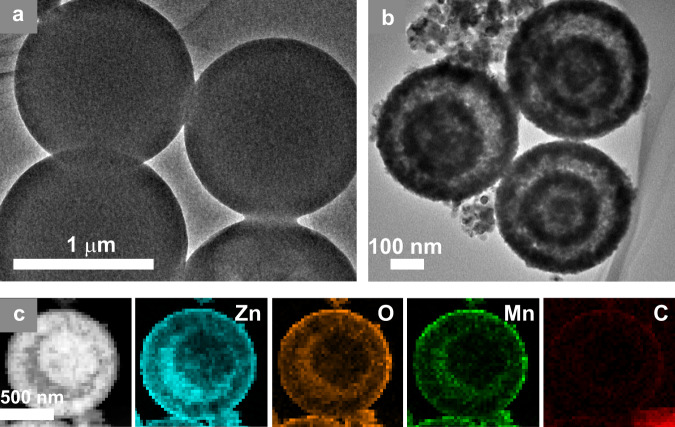
Morphology of the prepared materials. TEM images of the Mn, Zn-CPSs **a** before calcination and **b** after calcination for 3 h. **c** HAADF-STEM image of Mn, C-ZnO and EDS mapping of the sample show the constituent elements Zn, O, Mn, and C.

Transmittance electron microscopy (TEM) (Fig. 2) images show Mn, Zn-CPSs as high uniform solid spheres (Fig. 2a) with an average diameter of about 1.3 μm and smooth surface texture. After calcination for 3 h, unexpectedly, the solid Mn, Zn-CPSs were converted into unique concentric Mn, C-ZnO CTSHSs, as noticed from the high contrast between the shell edges and centered hollow regions (Fig. 2b). The CTSHSs retain the spherical shape of the CPS precursor but the diameter is dramatically shrunk to 650 nm due to the contraction upon calcination. The surface turns rough, pervaded with crystallites with invasive pores. The size of the crystallites was measured to be 25 nm from high-resolution TEM image. Its porous structure was also confirmed by N_2_ adsorption–desorption isotherm (Supplementary Fig. 2 and Supplementary Table 1). Additionally, uniform distribution of the constituent elements ca. Zn, O, Mn, and C are observed from energy dispersive X-ray spectroscopy (EDS) elemental mapping of the calcined sample (as taken for sample calcined for 1h, Fig. 2c). The elemental distribution is presented in Supplementary Fig. 3 and the actual compositional percentages of Mn and C in 2% Mn, C-ZnO sample are 1.8 and 8.3%, as derived from elemental analysis and inductively coupled plasma atomic emission spectroscopy measurements, respectively.

**Fig. 3:**
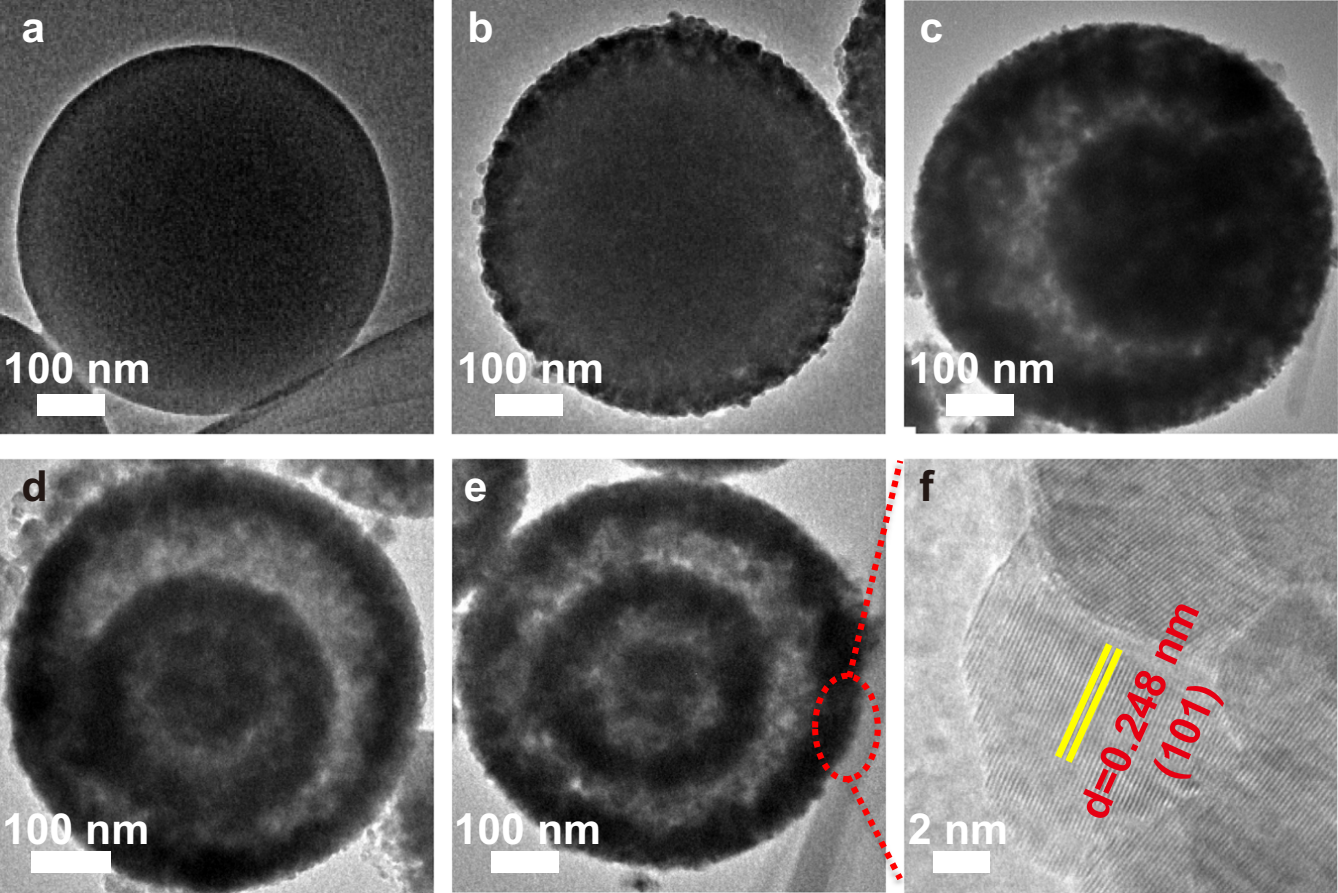
TEM images of Mn, Zn-CPSs. **a** Before calcination and after calcination for **b** 0.5  h, **c** 1  h, **d** 2  h, and **e** 3 h. **f** Depicts the HRTEM of Mn, C-codoped ZnO with lattice fringes of 0.248 nm corresponds to the (101) plane of Wurtzite ZnO.

To elucidate the evolution mechanism of the CTSHS morphology, TEM and field emission scanning electron microscopy (FESEM) images of the intermediates at different calcination stages were recorded (Fig. 3 and Supplementary Fig. 4). Before calcination, the solid spheres with distinct boundary are observed (Fig. 3a). At the initial stages of the calcination process (after 0.5 h), a significant temperature gradient (∆*T*) along the radial direction of the CPSs was generated where the outermost building units are subjected to abrupt heating. Meanwhile, the carbon entities vanish and ZnO nanoparticles were crystallized at the outer surface. Thus, a core–shell structure (the first ZnO shell conjoined to the entire core) was generated (Fig. 3b). Once forming, the rigid ZnO shell plays an indispensable role in preventing further contraction of the outer diameter, even if the entire core suffers from continuous shrinkage upon the disintegration of organic moieties by further annealing, known as heterogeneous contraction^31–34^.

With extended calcination time, both the core and the emerged new shell experienced two opposite forces. Namely, inward cohesion force (*F*_c_) originated from the sharp contraction of the interior core upon continuous disintegration of the skeletal carbon and outward adhesion force (*F*_a_) emanated from the cling of adjacent crystallites. The balance between both forces culminated in varied structures. When the CPSs were calcined for 1 h, the temperature gradient gets smaller. And *F*_c_ exceeds *F*_a_, which leads to separation of the entire core from the outer shell, forming a yolk-shell structure (Fig. 3c and Supplementary Fig. 4b). With calcination continued for 2 or 3 h, the entire yolk underwent similar processes described before, forming the unique second (core–double shell, Fig. 3d and Supplementary Fig. 4c) and the third shell (core–triple shell, Fig. 3e and Supplementary Fig. 4d). It is noteworthy that the disintegration of the carbon moieties on both exterior and interior surfaces induces the formation of observable pores within the shell layers (Supplementary Fig. 4e), which facilitates the mass transfer of the reactants/products^35,36^.

All the calcined samples together with commercial ZnO (for comparison) show diffraction peaks (Supplementary Fig. 5) that are well indexed to Wurtzite hexagonal ZnO (JCPDS PDF Card No. 36-1451), which are in line with the (101) lattice fringes of ZnO (Fig. 3f)^37^. Adequate purity and good crystallinity of the prepared ZnO CTSHS samples are reflected from the sharp peaks and absence of secondary peaks even with 5% Mn loading. Additionally, compared with commercial ZnO, the lattice of the prepared catalysts is mostly contracted after the doping process (see Supplementary Note 1 for more discussion). These results suggest the successful doping of Mn and/or C within the crystal lattice of ZnO and Mn species are implemented to substitute Zn ions in the lattice.

### CO_2_ PR activity of resultant samples

**Fig. 4:**
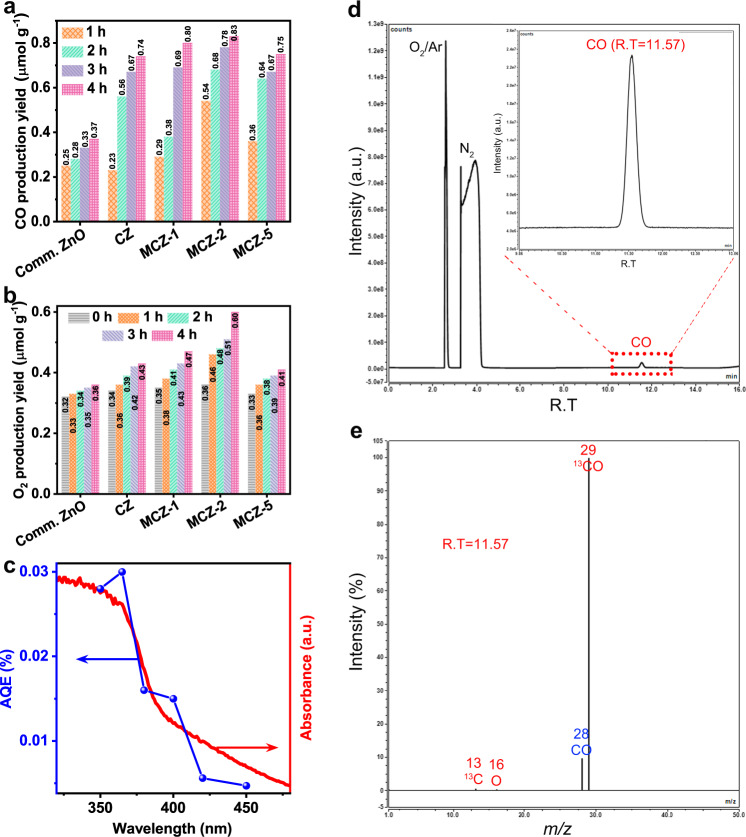
The photoresponse of the prepared photocatalysts. Photocatalytic CO_2_ reduction performance of comm. ZnO and the prepared samples: time course of **a** CO and **b** O_2_ production yields. **c** Wavelength dependence of the AQE and the UV–vis absorption spectrum of 2% Mn, C-ZnO sample. **d** Isotopic labeling chromatogram using ^13^NaHCO_3_ as a carbon source and **e** GC-MS data of the peak at retention time (RT) of 11.57 coressponding to ^13^CO.

*The photocatalytic performance of comm*. ZnO, CZ, and MCZ-*x* (CZ represents C-ZnO CTSHS sample, MCZ-*x* represents Mn, C-ZnO CTSHS samples with different percentages of Mn (*x* = 1, 2, and 5)) was evaluated in an online closed gas-circulation system under simulated solar light with a Quartz and Pyrex glass hybrid reaction cell (Supplementary Fig. 6). Ambient conditions were kept, and neither photosensitizer, precious cocatalyst nor sacrificial reagents were used. Carbon monoxide (CO) was the predominant reduction product, whereas oxygen (O_2_) was the oxidation product (Fig. 4). The 2% Mn, C-ZnO CTSHS photocatalyst (MCZ-2 in Fig. 4a) afforded the highest PR activity with CO yield rate of 0.83 μmol g^–1^. When Mn loading is too high, *i.e.* 5%, excessive defect states are introduced into the lattice of ZnO, which act as local recombination centers for photogenerated charge carriers^38^. H_2_ gas was hardly detectable, suggesting that the competitive H_2_ evolution from water is inhibited without the help of noble metal cocatalyst. The initial detected O_2_ gas came from the input high-purity CO_2_. As the CO_2_ PR proceeded, the amount of O_2_ increased gradually, indicating that water was photooxidized to O_2_ (Fig. 4b). Taking into account the sluggish kinetics of water oxidation and the need of four hole equivalents to produce one oxygen molecule^39^, it is reasonable to observe a lower yield of O_2_ (oxidation product) than CO (reduction product).

The apparent quantum efficiency (AQE) of CO is defined by the ratio of the effective electrons used for CO production to the total input photon flux^40,41^. It was measured by applying a 300 W xenon arc lamp with monochromatic light band-pass filters (*λ* = 350, 365, 380, 400, 420, and 450 nm; Beijing Perfectlight, China). The measured light intensity per unit area (*E*), the CO production yield (*M*_CO_), and the calculated CO production rate (*R*_CO_), AQE at different monochromatic wavelengths over the 2% Mn, and C-ZnO sample are shown in Table 1. Further, the wavelength dependence of the AQE and the UV–vis absorption spectrum of 2% Mn, C-ZnO sample is shown in Fig. 4c. Clearly, the trends of AQE match well with the light absorption spectrum, indicating the photocatalytic feature of the CO_2_ reduction reaction.

**Table 1:** The measured *E*, *M*_CO_, and the calculated *R*_CO_, AQE at different monochromatic wavelengths over the 2% Mn, C-ZnO sample.

*λ* (nm)	350	365	380	400	420	450
*E* (mW cm^–2^)	5	7	8	7	14	12
*M*_CO_ (ppm)	48	75	48	42	33	25
*R*_CO_ (μmol h^–1^)	0.033	0.051	0.033	0.029	0.022	0.017
AQE (%)	0.028	0.030	0.016	0.015	0.0056	0.0047

The recyclability and stability of MCZ-2 for photocatalytic CO_2_ reduction were also investigated (Supplementary Fig. 7). After four cycles, the decay of CO production yield is hardly perceptible. Meanwhile, the recycled photocatalyst shows identical phase composition as the fresh sample as confirmed from X-ray diffraction (XRD) results (Supplementary Fig. 8). These findings unambiguously disclosed the promising potential of the prepared CTSHS photocatalysts for CO_2_ PR.

Control experiments showed that no products were detected in the absence of photocatalysts and/or CO_2_ either in dark or under light irradiation, suggesting that CO is exclusively generated from CO_2_ PR. We performed isotopic labeling experiment using ^13^CO_2_ as isotopic tracer to investigate the carbon source of the obtained products. As shown in Fig. 4d, the total ion chromatographic signal around 11.57 min corresponds to CO, which produces three signals in the mass spectra (Fig. 4e). The main MS signal at *m*/*z* = 29 belongs to ^13^CO and the others at *m*/*z* = 13 and *m*/*z* = 16 are assigned to the fragments of ^13^CO, i.e. ^13^C and ^16^O, respectively. This result confirms that CO is indeed derived from CO_2_ reduction over MCZ-2 rather than any other carbon source^42,43^. In addition, the total ion chromatographic signals around 2.67 and 3.55 min are assigned to O_2_ and N_2_, respectively (Supplementary Fig. 9).

### Photocatalytic mechanism for enhanced performance

**Fig. 5:**
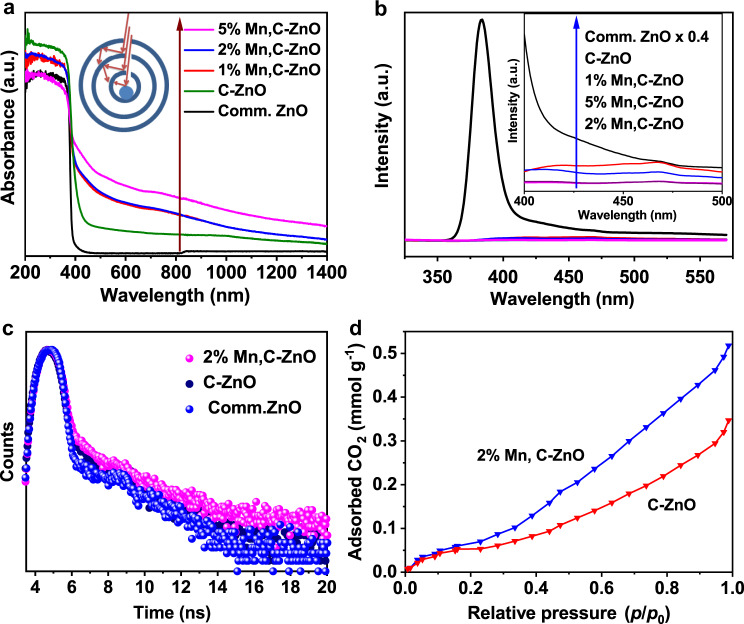
Optical and adsorption properties studied catalysts. **a** UV–vis light absorption of the prepared photocatalysts and the inset represents the multiple reflection effect of the incident light inside the hollow cavities. **b** PL spectra of photocatalysts at excitation wavelength of 380 nm. **c** TRPL spectra of the samples. **d** CO_2_ adsorption isotherm of C-ZnO CTSHSs and 2% Mn, C-ZnO-CTSHS samples.

Appreciable enhancement of the visible light absorption was sustained by the fascinating hollow morphology, thanks to the multiple reflections of the incident light inside the interior cavities of the fascinating core–triple shell hollow structure^44^ (inset of Fig. 5a). Moreover, the codoping process introduces impurity levels within the forbidden gap of ZnO, which indeed contributes to narrowing the bandgap (as depicted from Tauc plots, Supplementary Fig. 10) and enhancing the light-harvesting ability of the doped samples. A stepwise improvement of the light absorptivity of the prepared photocatalysts is observed in line with Mn-content (Fig. 5a). This finding should be correlated to the fact that more impurity states are developed within ZnO bandgap with increasing Mn doping. In addition, the doping with Mn could initiate other electronic transitions such as ligand to metal charge transfer, metal to ligand charge transfer, and d–d transition^45–47^. All these transitions are accounted for the whole system light harvesting, which leads to enlarged absorption response^48,49^.

Furthermore, the photoluminescence (PL) spectra of the prepared catalysts are step-wisely quenched after Mn and C doping. This reveals that suppressed charge recombination is achieved by doping. The 2% Mn, C-ZnO sample exhibits the lowest PL behavior (Fig. 5b), benefiting from two factors. One is (i) the defect state. The rest is (ii) the core–shell morphology and porous structure. The former could act as trapping centers, quenching the electron–hole inhalation process. Whereas the latter reduces both diffusion lengths of charges and reactants, thus minimizing charges recombination opportunities and providing accessible channels for mass/charge transfer^50^. Time-resolved photoluminescence (TRPL) results also demonstrate that 2% Mn, C-ZnO can retard charge carriers recombination and prolong their lifetime (average charge carrier-lifetime ca. 1.1, 0.9, and 0.8 ns for 2% Mn, C-ZnO, C-ZnO, and Comm. ZnO, respectively, Fig. 5c and Supplementary Table 3). These results indeed affirm the potential of Mn doping to delay charge recombination.

In addition, the prepared samples (C-ZnO and Mn, C-ZnO CTSHSs) attain very low resistance for charges transfer (*R*_ct_) process compared to comm. ZnO sample (as speculated from the electrochemical impedance spectroscopy measurements, Supplementary Fig. 11, where smaller arc diameter represents lesser resistance). Obviously, the fascinating architecture favors charge carrier separation and diffusion from bulk to surface region through few nanometer-thick shells. Especially, the Mn-doped sample shows the least *R*_ct_, benefiting from charge trapping action exerted by the doping-based defect states (at optimum content) and the following interfacial charge transfer^51,52^.

Besides, Mn species could be beneficial for enhancing the adsorption of CO_2_. To authenticate such a hypothesis, the CO_2_ adsorption isotherms for C-ZnO and 2% Mn, C-ZnO-CTSHSs are provided in Fig. 5d. Obviously, the 2% Mn-doped sample shows much more adsorption affinity (merely twice) toward CO_2_ than the undoped sample does. This result coincides well with the photocatalytic activity of both samples. The higher the CO_2_ adsorption affinity of a photocatalyst is, the better the PR activity will be expected. Therefore, it is reasonable to notice such enhanced activity for Mn-doped photocatalyst. The improvement of CO_2_ adsoprtion after Mn doping was also reported for Mn-In_2_S_3_ tested for CO_2_ electroreduction^53^.

Two plausible factors contributing to the high CO_2_ adsorption affinity are proposed. One is the higher content of surface oxygen-species (see XPS interpretation, Supplementary Note 2 for details) than C-ZnO sample. The other is the potential of Mn species replaced Zn ions in the lattice to activate CO_2_ molecules.

**Fig. 6:**
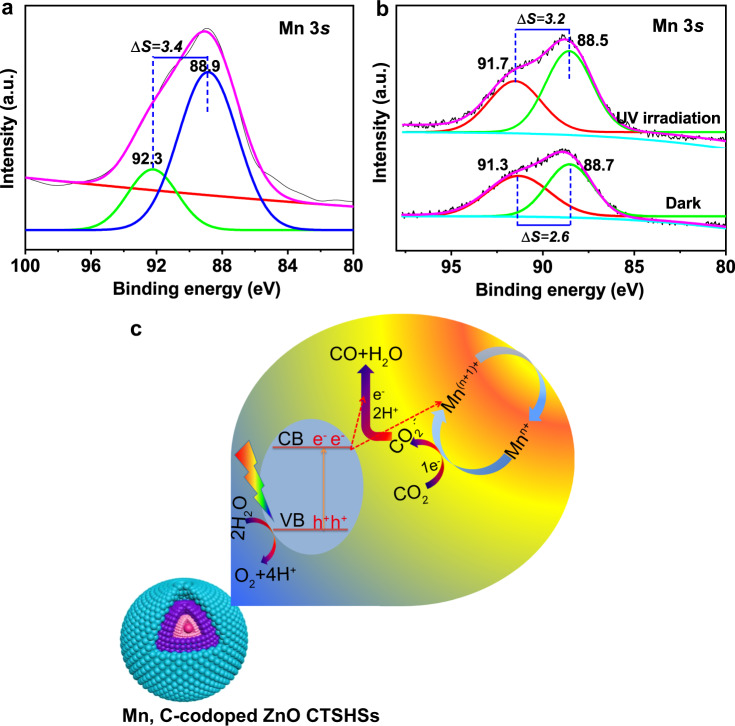
Interpretation of the CO_2_ reduction mechanism over Mn, C-ZnO photocatalyst. In situ XPS analysis showed Mn 3*s* profiles for **a** untreated and **b** CO_2_-adsorbed Mn, C-ZnO CTSHS samples. The multiple splitting of Mn 3*s* (∆*S*) is used to calculate average oxidation state of Mn ions within the structure (Eq. (1)). **c** Schematic illustration of CO_2_ activation and reduction over Mn, C-codoped ZnO sample. The CO_2_ molecules are firstly activated at the confined Mn active centers to the highly active radical anion. Such intermediate anion is then subjected to successive electron-coupled proton transfer process (electrons from ZnO CB and protons from water) and finally produces CO. The photogenerated holes are consumed for water oxidation (oxygen evolution reaction).

To further explore the functional role of Mn, in situ irradiated XPS (ISI-XPS) analysis for Mn, C-ZnO CTSHS sample saturated with CO_2_ was conducted under both dark and irradiated conditions. The high-resolution XPS peaks are demonstrated in Fig. 6 and Supplementary Fig. 12 together with the same peaks from bare sample (without CO_2_ treatment). The XPS spectra further indicate the successful doping of Mn within the crystal lattice of ZnO, originating from the distinctive Mn 2*p* and Mn 3*s* peaks appeared in the XPS profiles and the shift of Zn 2*p*_3/2_ peak (see Fig. 6 and Supplementary Fig. 12 for more details about the chemical state of Mn within the ZnO lattice). The presence of Mn is believed to promote catalytic conversions owing to their ability to share electrons with reactant molecules and/or intermediates^54–57^. Besides, doping gives rise to defect states and impurity levels. They not only reduce the bandgap of the semiconductor but also improve the light absorptivity of the photocatalyst. The defect states could support the charge separation process through obstructing the electron–hole recombination by electron trapping^58–61^. Specifically, introduced transition metal ions could act as distinctive active sites or enrich present active sites, which evidently support reactants’ adsorption, activation, and further chemical conversion^62–64^.

The loading of CO_2_ molecules onto the surface was assured by emergence of a tiny M-CO_3_ peak in C 1*s* XPS (Supplementary Fig. 12d), suggesting the successful bonding of CO_2_ molecules to metallic sites. To probe how Mn ions could assist activation of CO_2_ molecules during the PR process, we calculated the average oxidation states (AOS) of Mn under test according to the following equation^65^:

1 $${{{{{\rm{AOS}}}}}}=8.95-1.13\,\Delta S$$

where ∆*S* is the multiple splitting of Mn 3*s* peak (Fig. 6a, b). By substituting ∆*S* values in Eq. (1), we get a unit increase in the AOS for sample saturated with CO_2_ and kept in darkness. Then, a decrease in AOS by half a unit is observed for sample saturated with CO_2_ and irradiated with light. The significant increase in AOS of Mn after CO_2_ loading and before illumination could be explained in terms of electron transfer from Mn centers to adsorbed CO_2_ molecules^54,55^. In our system, we noticed that the primal AOS of Mn in CO_2_-loaded photocatalyst can be restored again under light irradiation. This is due to photogenerated electrons transfer from ZnO-conduction band (CB) to Mn^(*n*+1)+^ centers to regenerate the primal Mn^n+^ again (Fig. 6c). This light-switchable conversion between Mn^*n*+^/Mn^(*n*+1)+^ is very critical for restoring the original activity of the Mn-doped catalyst without external treatment or activation steps. In addition, this conversion holds a promising potential for all-in-one CO_2_ reduction technology. Concisely, Mn^(*n*+1)+^ species can capture the photogenerated electrons from the CB of ZnO, yielding Mn^*n*+^, which functions as the active centers for CO_2_ adsorption and activation via one electron-transfer process to yield CO_2_·^–^ and Mn^(*n*+1)+^. At the same time, if more electrons are captured, the generation of CO becomes possible. This finding authenticates the role of Mn species as ionized cocatalyst in the system. Because we measure the change of AOS, further investigations are required to elucidate the actual functional oxidation state involved in CO_2_ activation.

In fact, the regeneration of the Mn species after the activation step is very critical to maintain the activity. Moreover, the charge trapping action is of great essence for charge carrier’s separation. Thus the optimal doping from Mn results in lowest charges recombination as revealed from PL results.

In this vein, Mn functions as Lewis base center, which supports the adsorption of acidic CO_2_ molecules and further transfer of one electron to culminate activated CO_2_·^–^ (Fig. 6c). Such a step is followed by proton-coupled electron-transfer process to produce the desired molecules (Supplementary Eqs. (1)–(7)).

The adsorption, activation and subsequent reduction of CO_2_ molecules over Mn-doped ZnO photocatalyst are further confirmed by in situ diffuse reflectance infrared Fourier transform spectroscopy (DRIFTS) results (Supplementary Fig. 13 and Supplementary Note 3), which assure the adsorption, activation, and successful conversion of activated CO_2_ molecules onto CO at the photocatalyst surface (Fig. 6c).

All these results unambiguously disclosed the potential of the prepared and light-switchable ionized cocatalyst (Mn^*n*+^/Mn^(*n*+1)+^) integrated C-ZnO CTSHSs as an efficient photocatalyst for CO_2_ PR under ambient conditions without sacrificial reagents, precious cocatalysts, or external treatment set up for activity restoration.

In summary, core–triple shell Mn, C-codoped ZnO hollow spheres were rationally fabricated *via* a one-pot synthetic protocol. In addition, they were tested as a highly active and selective catalyst for CO_2_ PR under simulated sunlight and ambient conditions. The unique core–triple shell hollow structures codoped with Mn and C hold manifold structural and functional features. These unique features culminated improved light absorption capability, enhanced charge separation and migration efficiency, suppressed e^−^/h^+^ pairs undesired recombination probability and abundant adsorption and activation active sites for effective photoredox catalysis. All of these accounted for the superior activity of the prepared photocatalyst. The Mn species played a significant role to activate CO_2_ molecules through an electron-transfer mechanism. We have observed that the ionized Mn species can restore their primal oxidation state and hence their activity by virtue of the photogenerated electrons at the ZnO CB. Therefore, it can be operated as a light-switchable ionized cocatalytic system. These findings will support the design of efficient photocatalytic materials for sustainable clean energy production and beyond. The present study sheds light on the significance of hollow structure materials as an active photocatalyst for CO_2_ reduction as well as the importance of light-switchable ionized cocatalysts during this process.

## Methods

### Materials

All chemicals are of analytical grade and used without additional purification. Zinc acetate dihydrate (Zn(CH_3_COO)_2_·2H_2_O) and absolute ethanol (EtOH) were purchased from Beijing Sinopham Chemical Reagent Co., Ltd. Manganese acetate tetrahydrate (Mn(CH_3_COO)_2_·4H_2_O)) and salicylic acid (C_7_H_6_O_3_) were purchased from Tianjin BODI Chemical Co., Ltd. PVP (C_6_H_9_NO)*n* (K23-27, MWt ~24,000) was supplied by Aladdin Industrial cooperation, Shanghai, China.

### Synthesis of Mn, C-ZnO CTSHSs

The Mn, Zn-CPSs were firstly prepared by a solvothermal step. Typically, 1 mmol of Zn(CH_3_COO)_2_·2H_2_O, *x* mmol of Mn(CH_3_COO)_2_·4H_2_O corresponding to the desired Mn^2+^/Zn^2+^ mole ratio (0, 0.01, 0.02 or 0.05), and (2 + *x*) mmol of salicylic acid were magnetically dissolved in 50 mL of EtOH. Afterward, a calculated amount of PVP (to keep the concentration ratio *M*_PVP_/*M*_M_^2+^ = 5, *M* of PVP was calculated based on the molecular weight of its monomer) was added to the above solution and further stirred for 30 min. The mixture was transferred into a 100 mL of Teflon-lined stainless steel autoclave, heated at 160 °C for 12 h, and then air-cooled to room temperature. The precipitate was separated by centrifugation, washed three times with EtOH, and dried under vacuum at 60 °C overnight. The as-obtained Mn, Zn-CPSs were calcined at 550 °C for 3 h by 5 °C/min rate under air atmosphere. The samples with 0, 1, 2, and 5% Mn were denoted as C-ZnO (CZ), 1% Mn, C-ZnO (MCZ-1), 2% Mn, C-ZnO (MCZ-2), and 5% Mn, C-ZnO (MCZ-5), respectively.

### Characterization

TEM, HRTEM, and elemental mapping were obtained by Titan G2 60–300 operated at 300 kV. FESEM images were taken by JSM-7500F, JEOL, Japan, equipped with X-Max 50 EDS unit (Oxford Instruments, UK). XRD patterns of the prepared samples were recorded using D/Max-RB X-ray diffractometer (Rigaku, Japan) with Cu *K*α radiation (*λ* = 1.5406 Å) and scan rate = 0.05 ° S^–1^. The surface composition and chemical states of C-ZnO and 2% Mn, C-ZnO CTSHS samples were analyzed by XPS analysis (Thermo ESCALAB 250). ISI-XPS analysis for 2% Mn, C-ZnO CTSHS sample saturated with CO_2_ was conducted in darkness as well as under light irradiation to probe the oxidation state change of Mn during CO_2_ PR. The 2% Mn, C-ZnO CTSHS sample was firstly degassed at 150 °C for 4 h to remove pre-adsorbed species. Afterwards, it was saturated with CO_2_ by continuously purging with pure CO_2_ for 4 h prior to the XPS test. A 3 W 365 nm LED was used as the light source during ISI-XPS test. The UV–visible light absorption spectra of the samples were recorded by UV-2600, Shimadzu spectrophotometer (Japan) where BaSO_4_ was used as the reflectance standard. The Brunauer–Emmett–Teller specific surface area and porosity of the prepared samples, nitrogen adsorption–desorption isotherms were measured on a Micromeritics ASAP 2020 apparatus (USA) and the adsorption data at relative pressure (*p*/*p*_0_) range of 0.05–0.3 were used. The average pore size and pore volume of the samples were calculated through the Barret–Joyner–Halender method using the adsorbed nitrogen volume at the relative pressure of 0.994. PL spectra were recorded on a Hitachi F-7000 (Japan) fluorescence spectrophotometer. FLS1000 fluorescence lifetime spectrophotometer (Edinburgh, Instruments, UK) was employed to record TRPL spectra of the prepared samples.

FTIR and DRIFTS analyses were obtained by an FTIR spectrometer (Thermo Scientific, Nicolet iS50, USA). For DRIFT test, the prepared sample was placed into the reaction chamber, then the chamber was sealed and purged with the mixed CO_2_ and H_2_O gases. The DRIFTS analysis was conducted under both dark and light irradiation (3 W 365 nm LED) conditions.

The electrochemical measurements were performed in a standard three-electrode configuration analyzer (CHI660C instruments, CHI, China) system with Pt wire and Ag/AgCl (saturated KCl) utilized as counter and reference electrodes, respectively, while the working electrode was prepared using the as-synthesized samples deposited on FTO glass, and the three electrodes were immersed in 0.5 M Na_2_SO_4_ aqueous solution as electrolyte.

### Photocatalytic CO_2_ reduction and isotope-labeling measurement

The photocatalytic reduction of CO_2_ was conducted in an online gas-closed system with a gas-circulated pump. Typically, 50 mg of photocatalysts and 1 mL of H_2_O were added in a Quartz and Pyrex glass hybrid reactor connecting to the CO_2_ PR system (Supplementary Fig. 6). After complete evacuation of the reaction system, ~80 kPa of high-purity CO_2_ (99.999%) gas was injected into the air-tight system. After adsorption equilibrium, a 300 W Xe arc lamp (PLS-SXE300D, Beijing Perfectlight, China) was used as the light source. The photocatalytic CO_2_ reduction products were analyzed by a gas chromatograph (GC-2030, Shimadzu Corp., Japan) equipped with barrier discharge ionization detector (BID) and a capillary column (Carboxen 1010 PLOT Capillary, 60 m × 0.53 mm). The temperatures of the injector and BID were set to be 150 and 280 °C, respectively. The AQE of CO over the 2% Mn, C-ZnO sample was calculated as follows:

2 $${{{{{\rm{AQE}}}}}}( \% ) =\frac{{{{{{\rm{number}}}}}}\,{{{{{\rm{of}}}}}}\,{{{{{\rm{reacted}}}}}}\,{{{{{\rm{electrons}}}}}}}{{{{{{\rm{number}}}}}}\,{{{{{\rm{of}}}}}}\,{{{{{\rm{incident}}}}}}\,{{{{{\rm{photons}}}}}}}\times 100\\ =\frac{{{{{{\rm{number}}}}}}\,{{{{{\rm{of}}}}}}\,{{{{{\rm{CO}}}}}}\,{{{{{\rm{molecules}}}}}}\times 2}{{{{{{\rm{number}}}}}}\,{{{{{\rm{of}}}}}}\,{{{{{\rm{incident}}}}}}\,{{{{{\rm{photons}}}}}}}\times 100\\ =\frac{2\times {R}_{{{{{{\rm{CO}}}}}}}\times {t}_{1}\times {N}_{A}}{P\times {t}_{2}\times \frac{\lambda }{hc}}\times 100$$

where *R*_CO_ is the CO production rate (mol h^–1^); *t*_1_ is the irradiation time (1 h); *N*_A_ is Avogadro constant (6.02 × 10^23^ mol^–1^); *P* is the total incident light flux (W, J s^–1^) and equals light intensity per unit area (*E*, W cm^–2^) times effective irradiation area (*S*, cm^2^), where *E* can be measured by the radiant power energy meter (UV-A and FZ-A, Photoelectric Instrument Factory of Beijing Normal University), *S* is 4.5 cm^2^ in this experiment; *t*_2_ equals 3600 s; *λ* is the monochromatic light wavelength (m); *h* is the Planck constant (6.626 × 10^–34^ J s) and *c* is the light speed in vacuum (3 × 10^8^ m s^–1^).

^13^CO_2_ isotope tracer experiment was conducted to verify the carbon source of the products by using ^13^C isotope-labeled sodium bicarbonate (NaH^13^CO_3_, Cambridge Isotope Laboratories Inc., USA) and H_2_SO_4_ aqueous solution for the photocatalysis examinations. Typically, 20 mg of photocatalysts, 2 mM of [Ru^II^(bpy)_3_]Cl_2_·6H_2_O, 5 mL of triethanolamine, 30 mL of acetonitrile, and 1 mL of water were added in a Pyrex glass reaction cell. After 1 h of photocatalytic reaction, 1 mL of the mixed gas was taken out from the reactor and analyzed by gas chromatography-mass spectrometry (TRACE 1300 and ISQ 7000, Thermo scientific, USA) equipped with the column for detecting the product of ^13^CO (TG-BOND Msieve 5 A, 30 m × 0.32 mm × 30 μm, Thermo Scientific, USA). The column was maintained at 45 °C for 15 min and then heated to 180 °C at 30 °C min^–1^ and maintained for 1 min. The temperatures of the injector, ion source, and MS transfer line were set to be 200, 230, and 250 °C, respectively.

## Supplementary information

Supplementary Information

## Data Availability

The raw data of Figs. 4, 5, 6a, b, Supplementary Figs. 1, 2, 5, 7–13 and Supplementary Tables 1 and 3 generated in this study have been deposited in the figshare database [10.6084/m9.figshare.14904753]. Source data are provided with this paper.

## Source data

Source Data
