# Supplementary material for: Sustained CO2-photoreduction activity and high selectivity over Mn, C-codoped ZnO core-triple shell hollow spheres
Source: Nat Commun. 2021 Aug 16;12:4936. doi: 10.1038/s41467-021-25007-6 (PMC8368040; doi:10.1038/s41467-021-25007-6)
Supplement: Supplementary file 1 — Supplementary Information [file 41467_2021_25007_MOESM1_ESM.pdf]

Supplementary Information

Sustained CO_2-photoreduction activity and high selectivity over Mn, C-codoped ZnO core-triple shell hollow spheres

Sayed and Xu *et al*.

Supplementary Figures

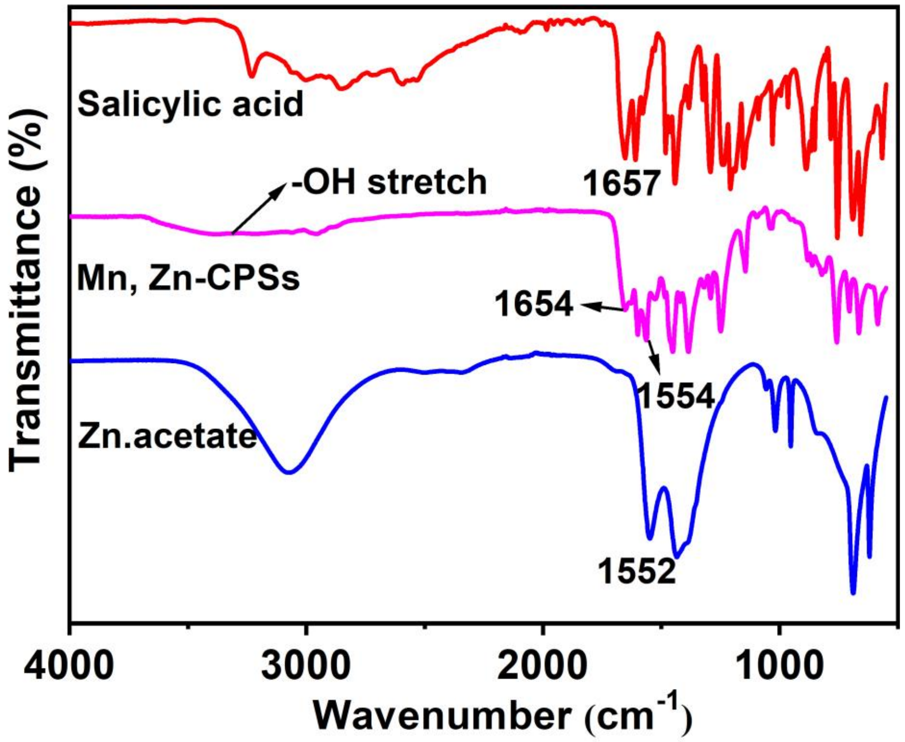

Supplementary Figure 1. FTIR spectra of salicylic acid, Zn acetate and Mn, Zn-CPSs.

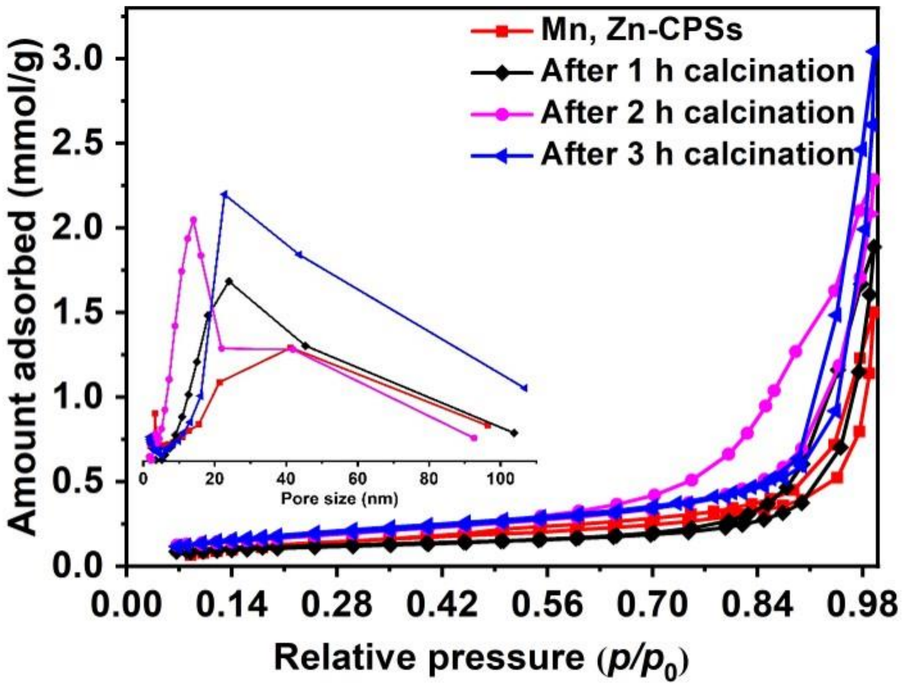

Supplementary Figure 2. N_2 adsorption-desorption isotherms of Mn, Zn-CPSs precursors, and the precursor after calcination for 1, 2 and 3 h. The inset represents the pore size distribution of the samples.

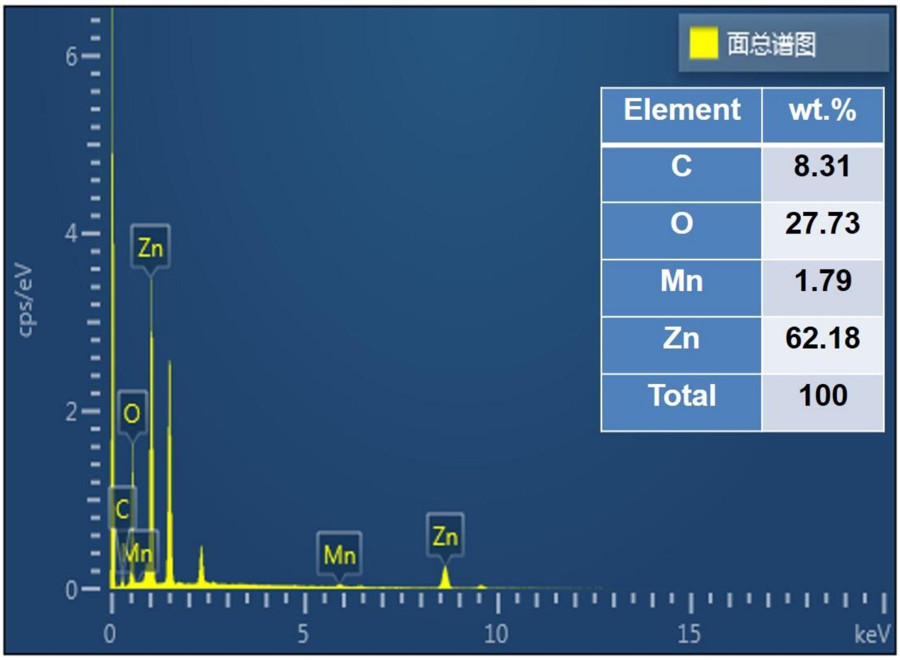

Supplementary Figure 3. EDS elemental signals, in which the inset table represents the weight percentage of each element in the sample.

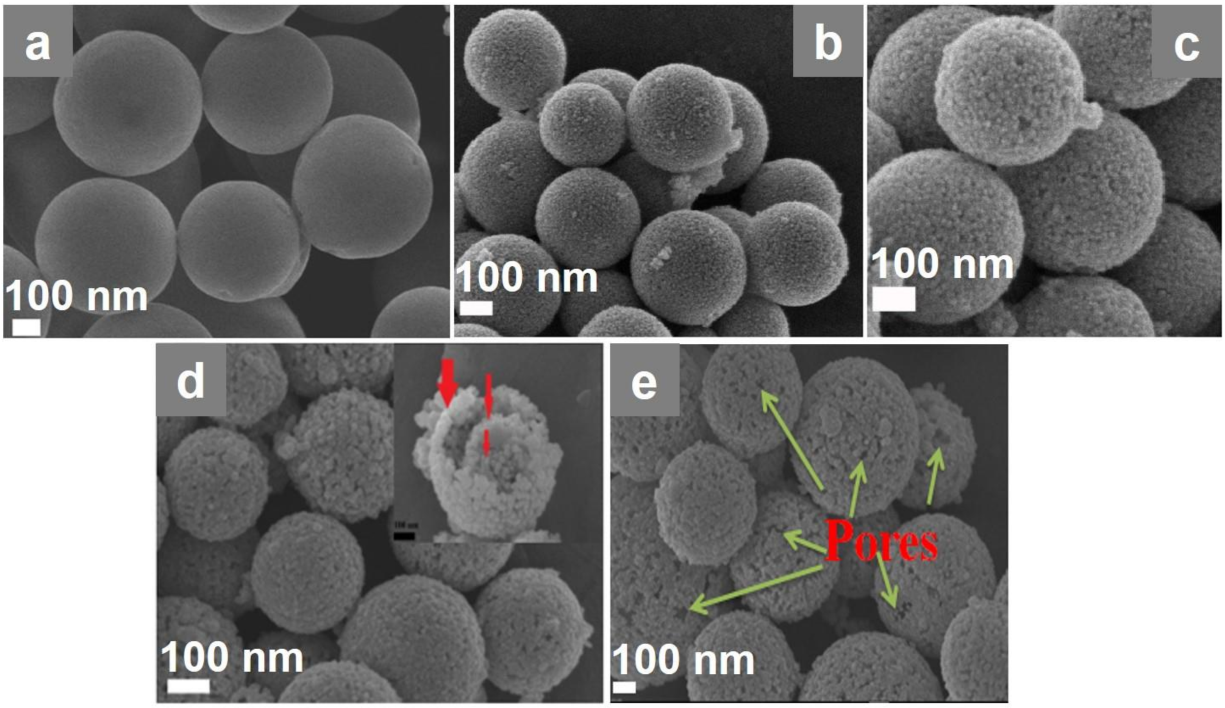

Supplementary Figure 4. Morphology of the prepared samples. FESEM of Mn, Zn CPSs precursors **a** before calcination, after calcination for **b** 1 h, **c** 2 h and **d** 3 h, the inset in **d** shows the core-triple shell morphology. **e** FESEM image shows the porosity of Mn, C-ZnO CTSHSs sample.

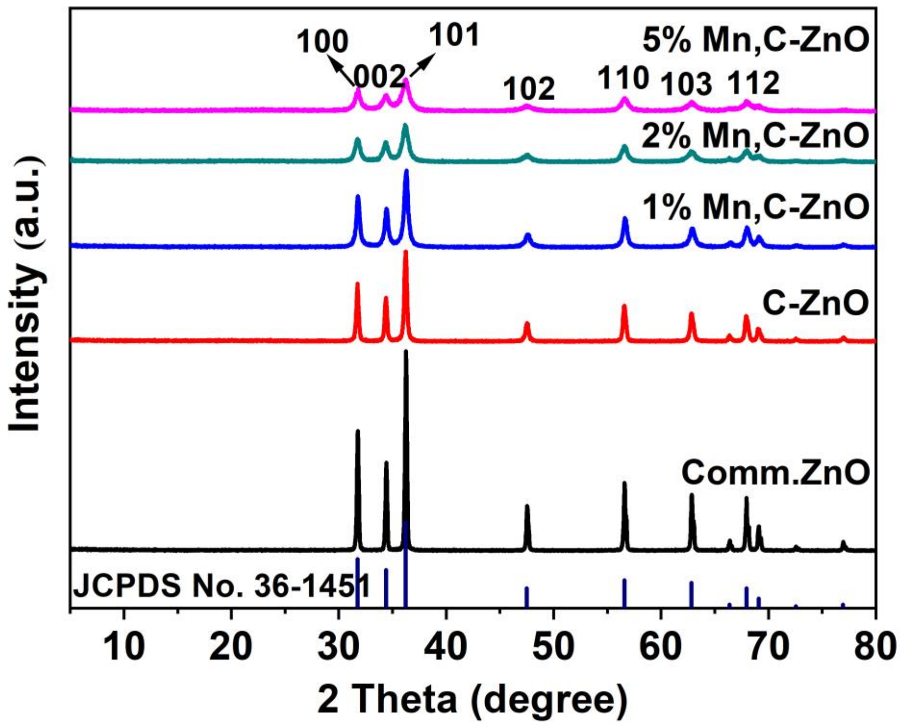

Supplementary Figure 5. XRD patterns of the prepared samples together with commercial ZnO.

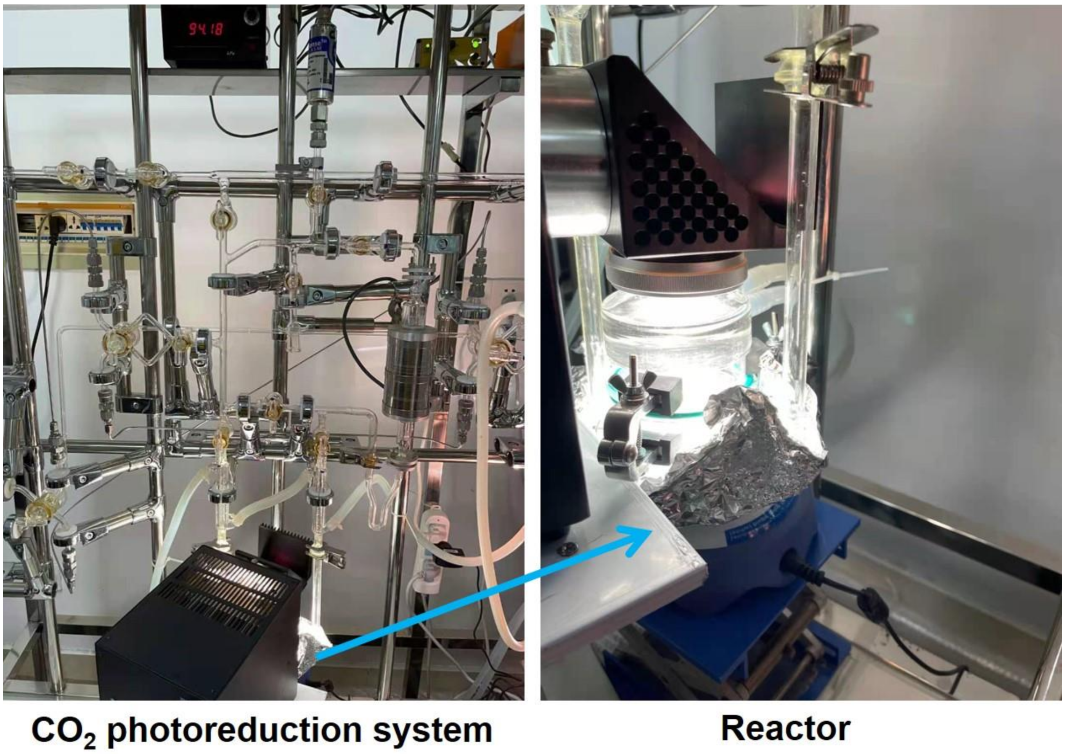

Supplementary Figure 6. The on-line closed-gas circulation system for CO_2 reduction.

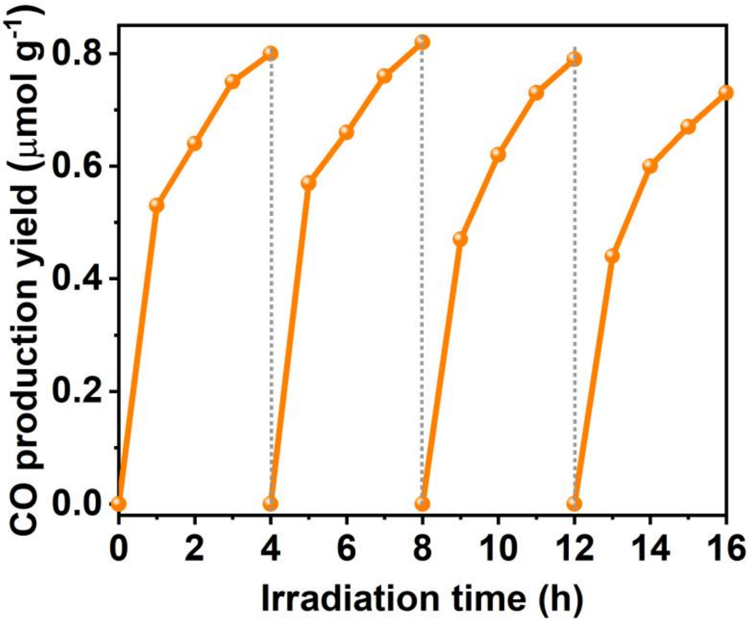

Supplementary Figure 7. The recyclability of 2% Mn, C-ZnO photocatalyst for the photocatalytic CO_2 reduction.

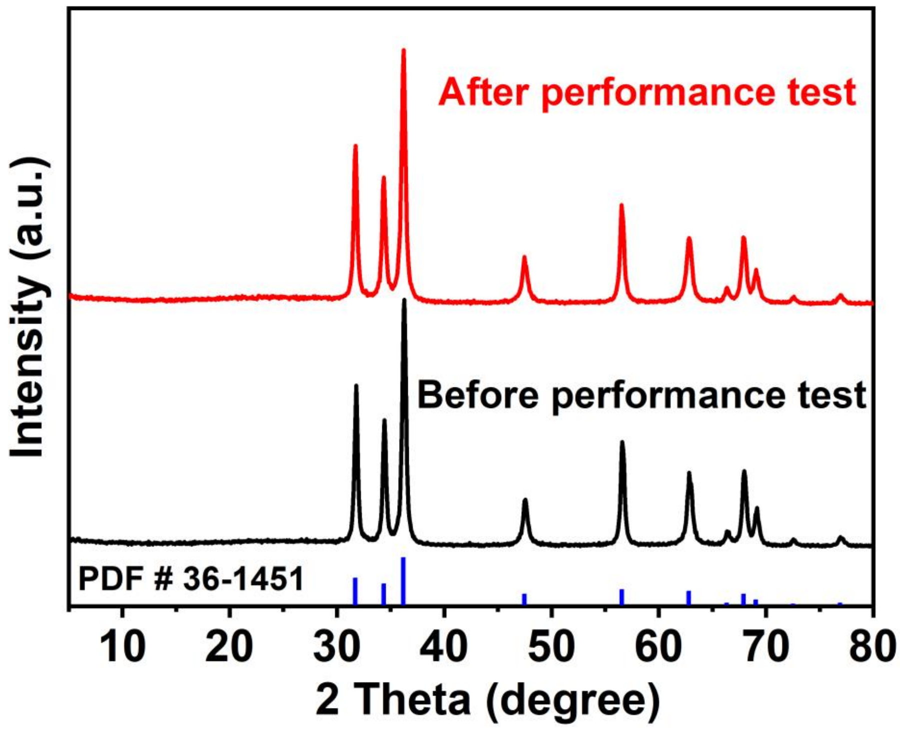

Supplementary Figure 8. XRD patterns of 2% Mn, C-ZnO CTSHSs before and after CO_2 reduction test. The results indicate the photocatalyst phase structure is consistent before and after the targeted reaction, which reflects its stability.

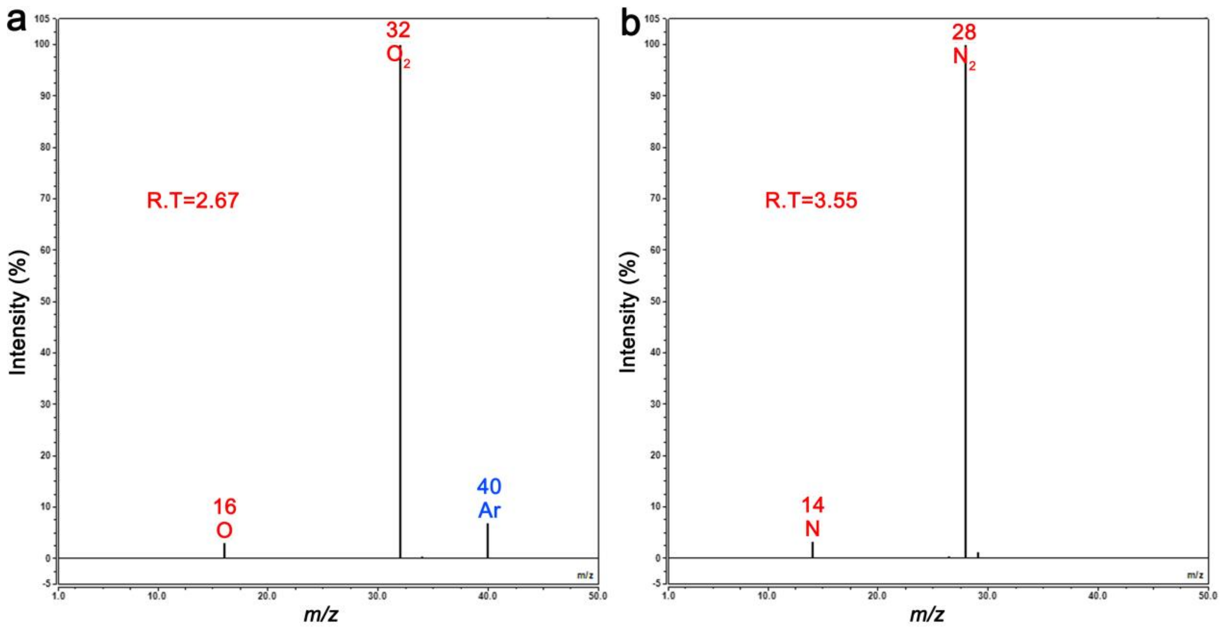

Supplementary Figure 9. GC-MS spectra of chromatographic peaks at retention time of **a** 2.67 min and **b** 3.55 min corresponding to O_2 and N_2, respectively.

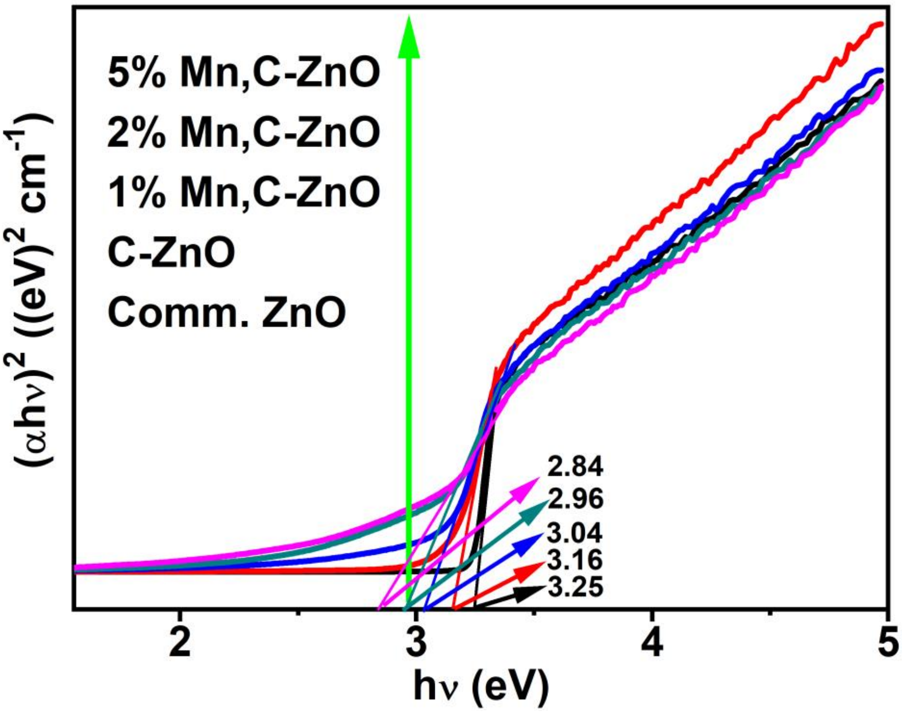

Supplementary Figure 10. Bandgap estimation of the prepared samples. Tauc plots of prepared C-ZnO and Mn, C-ZnO samples together with comm. ZnO. The results indicate a progressive narrowing of the ZnO-band gap after C and Mn doping.

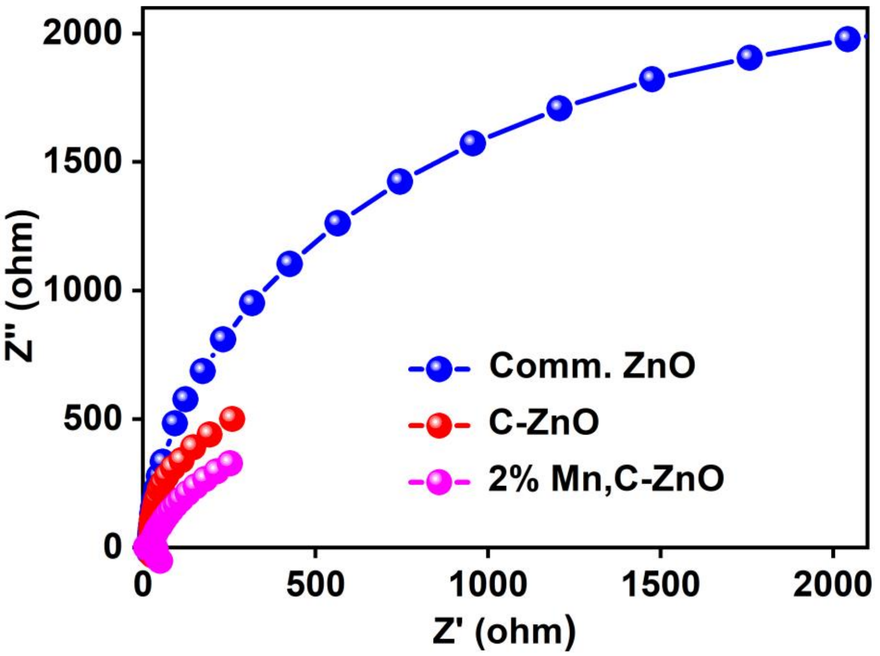

Supplementary Figure 11. Electrochemical impedance spectroscopy (EIS) results of the used catalysts, suggesting the smooth charge transfer in Mn, C-ZnO with the lowest charge transfer resistance (R_{ct}).

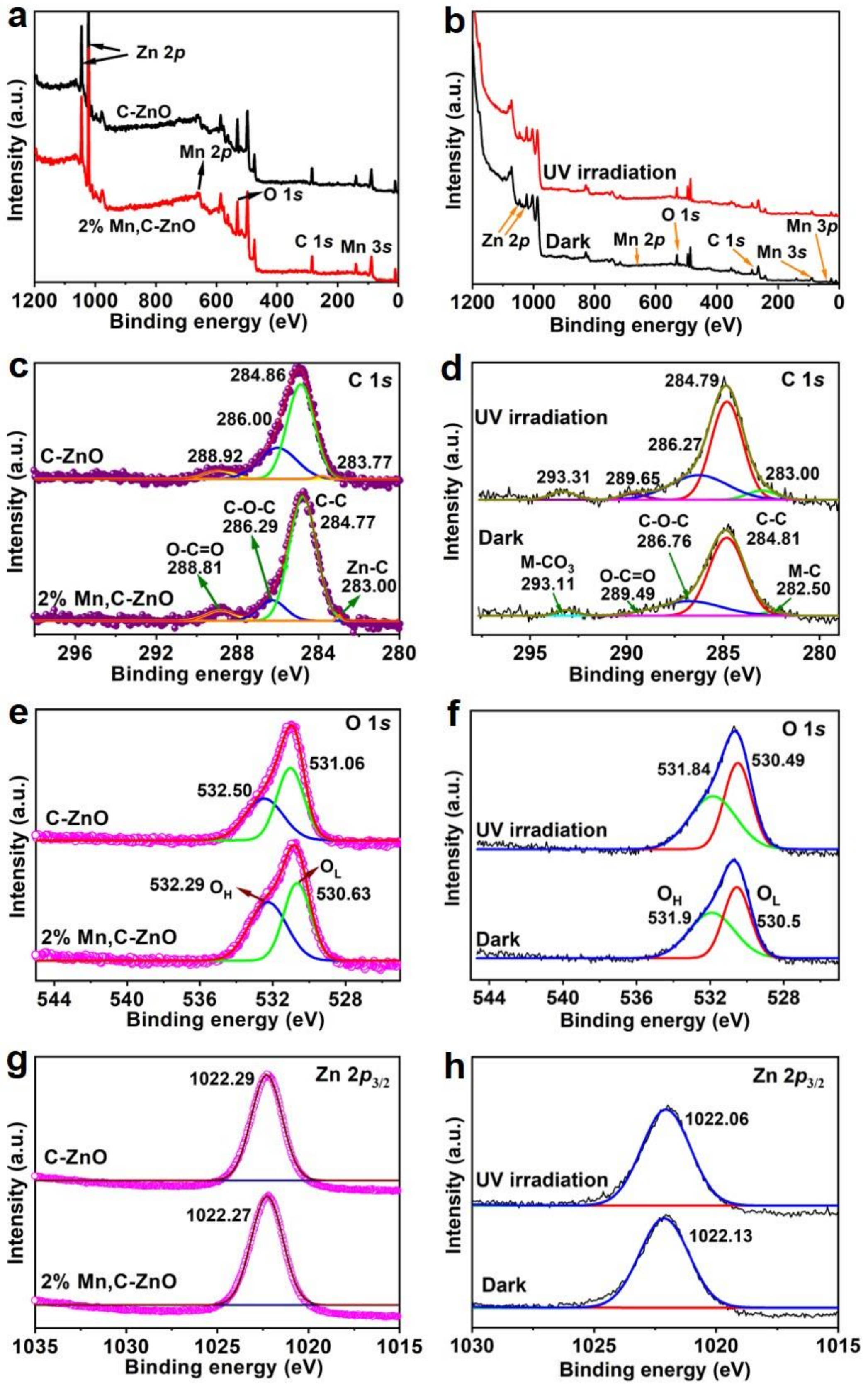

Supplementary Figure 12. Chemical analysis of samples. XPS spectra of C-ZnO and 2% Mn, C-ZnO CTSHSs samples (left panel) and 2% Mn, C-ZnO sample loaded with CO_2 under both dark and irradiation conditions (right panel). **a, b** The survey spectra of samples. The high resolution XPS of **c, d** C 1*s*, **e, f** O 1*s* and **g, h** Zn 2*p*.

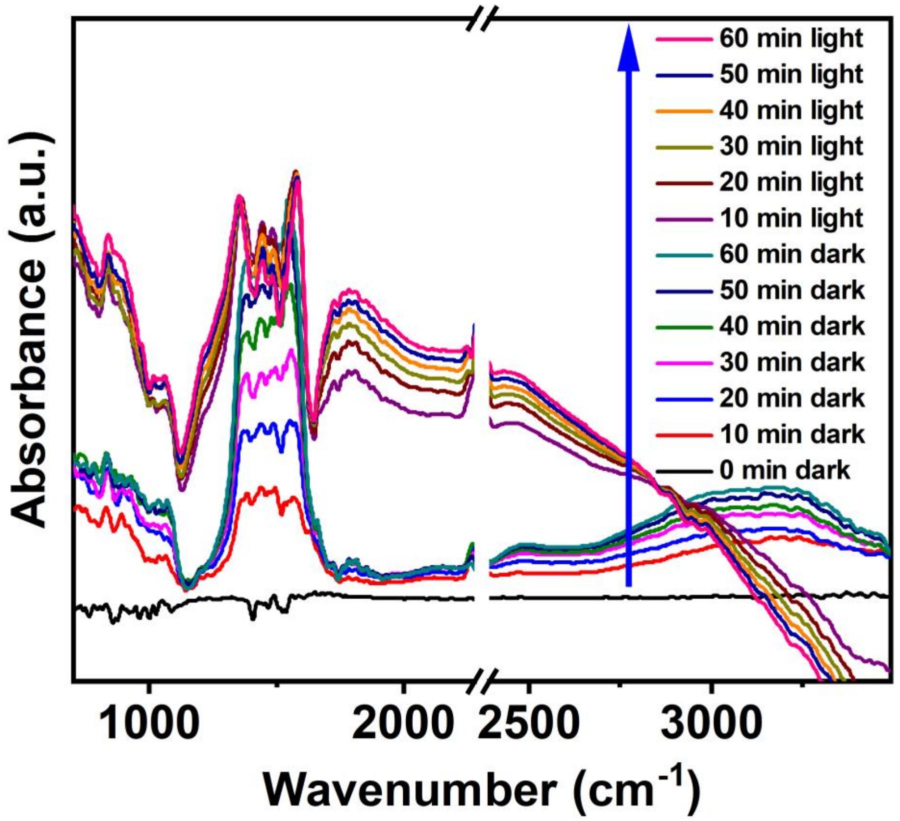

Supplementary Figure 13. CO_2 PR pathway over Mn, C-codoped ZnO photocatalyst. *In-situ* DRIFTS spectra of photocatalytic CO_2 reduction using 2% Mn, C-ZnO CTSHSs sample under dark condition and 365 nm LED light irradiation.

Supplementary Tables

Supplementary Table 1. BET surface area (S_{BET}), pore volume (V_{pore}) and average pore size (V_{pore}) of the starting Mn, Zn-CPSs and the calcined intermediate products.

Sample	S_{BET} (m^2 g^{-1})	V_{pore} (cm^3 g^{-1})	Average pore size (nm)
**Zn-Mn-CPSs**	5	0.03	28
**After calcination for 1 h**	9	0.05	29
**After calcination for 2 h**	11	0.05	19
**After calcination for 3 h (2% Mn, C-ZnO CTSHSs)**	15	0.11	28
**C-ZnO CTSHSs**	14	0.08	23
**Comm. ZnO**	14	0.02	7.5

Supplementary Table 2. XRD parameters of Mn, C-ZnO CTSHSs with different content of Mn together with Comm. ZnO.

Sample	Calculated* "a" (Å)	Calculated "c" (Å)	Diffraction from "100" plane		Diffraction from "101" plane	
			2 Theta	d (Å)	2 Theta	d (Å)
**Comm. ZnO**	3.2588	5.2518	31.648	2.8222	36.0992	2.4862
**C-ZnO**	3.2529	5.1024	31.7633	2.8152	36.3545	2.4692
**1% Mn, C-ZnO**	3.2502	5.0932	31.7802	2.813	36.1134	2.4852
**2% Mn, C-ZnO**	3.2526	5.236	31.7407	2.8171	36.1991	2.4794
**5% Mn, C-ZnO**	3.2466	5.2703	31.8209	2.8099	36.2408	2.4768

Supplementary Table 3. TRPL fitting parameters for the analyzed samples; comm. ZnO, C-ZnO and Mn, C-ZnO CTSHSs.

**Sample**	**τ_1 (ns) (percentage)**	**τ_2 (ns) (percentage)**	**B_1**	**B_2**	**τ (ns) (average)***
**Comm. ZnO**	0.25 (75%)	2.64 (25%)	9113	282	0.8
**C-ZnO**	0.17 (73%)	2.75 (27%)	7251	164	0.9
**2% Mn, C-ZnO**	0.24 (68%)	2.83 (32%)	8372	328	1.1

Supplementary Notes

Supplementary Note 1

XRD analysis. In **Supplementary Figure 5**, Obviously, (i) the absence of secondary phases arises from the doping process even with 5% Mn loading, (ii) dramatic change of the characteristic diffraction peaks and the apparent contraction on the cell parameters (**Supplementary Table 2**) of the prepared samples compared to those of typical hexagonal ZnO sample manifests that C and Mn were successfully doped to the ZnO lattice structure rather than attached to the surface. Mn could replace some Zn^{2+} within the ZnO lattice and may be introduced as Mn^{2+} or higher oxidation states. After the doping process, prepared ZnO CTSHSs samples exhibit lattice contraction (**Supplementary Table 2**, as indicated from the lattice constants), attributing to the fact that C ions with small ionic radius replaced the O ions within the lattice (ionic radii= 0.66, 0.58, 0.53, 0.46, 0.6, 0.29 and 1.24 Å for Mn^{2+}, Mn^{3+} , Mn^{4+}, Mn^{7+}, Zn^{2+}, C^{4-} and O^{2-}, respectively).[1] Based on this fact, it is reasonable to notice such a change in the lattice constants. Furthermore, crystal distortion and surface defects generated within the prepared samples upon C and Mn doping (further confirmed from XPS and DRS results) are very crucial for altering the surface energy of the photocatalysts and providing more active sites.[2]

The calculation of the lattice constants "a" and "c" is based on the following equation:[3]

$$\frac{1}{d^2} = \frac{4}{3}\left(\frac{h^2 + hk + k^2}{a^2}\right) + \frac{l^2}{c^2} \qquad (1)$$

where *h*, *k*, and *l* are Miller's indices. The values of "a" and "c" were derived from this equation by applying the values of the d-spacing of the (100) and (101) planes, respectively, (see **Supplementary Table 2**).

Supplementary Note 2

XPS interpretation. The XPS survey spectra of C-ZnO and 2% Mn, C-ZnO CTSHSs (untreated and CO_2-treated one under both dark and illumination conditions) samples are presented in **Supplementary Figure 12a and b**, which show the characteristic peaks for Zn, O, C and Mn. The high resolution XPS of Zn 2$p_{3/2}$ at ~ 1022 eV indicates Zn^{2+} state of ZnO in all samples (Supplementary Figure 6g and h).[4] The slight chemical shift of this peak to lower binding energies (BEs) after Mn insertion and CO_2 loading ascertains the finite Mn substitution of Zn^{2+} in the lattice of ZnO and an added Zn-Mn binding energy and possible emergence of new Zn-CO_2 bond, respectively, which are also confirmed from C 1*s* peak fitting.[5,6]

The XPS for the O 1*s* in all samples shows two different oxygen states. That (i) at higher BE is typically associated with loosely bound oxygen (O_H) on the surface such as OH groups and O^{2-} ions in oxygen-deficient regions. That (ii) at lower BE is due to lattice oxygen (O_L) from ZnO.[7–11] Obviously, the intensity of O_H peak increases upon Mn incorporation, indicating more surface oxygen-containing groups. Oxygen-containing groups are believed to not only facilitate the adsorption and activation of reactant molecules at the photocatalyst surface but also suppress the charge carrier recombination.[12] Therefore, it is reasonable to predict a higher photocatalytic activity of Mn, C-ZnO TSHSs samples over C-ZnO one. Additionally, the O_L peak has been shifted to higher BE after Mn doping revealing Mn-O-Zn bond.[7]

The core level C 1*s* XPS for all samples are fitted into four peaks and presented in **Supplementary Figure 12b and c**. The peaks around 288.9, 286.0, 284.8 and 283.0 eV are ascribed to C=C, C-O-C, C-C and M-C, respectively. Those were originated from hydrocarbon residues of the decomposed carbon skeleton and successful carbon doping.[13,14] After CO_2 treatment, a new tiny peak at ~293 eV evolved, which could be ascribed to M-CO_2 binding upon the interaction between metallic centers and loaded CO_2 molecules.

Supplementary Note 3

CO_2 reduction mechanism. Once activated at the preferential Mn active sites on the surface of the catalyst, the obtained $CO_2\cdot^-$ radical anions are highly reactive. They underwent several proton-coupled multiple-electron-transfer reactions, almost instantaneously, which culminated in a variety of valuable products.[2,15,16] To elucidate the photoreduction (PR) mechanism of CO_2 and explore the intermediate products of such process over 2% Mn, C-ZnO CTSHSs sample, *in-situ* DRIFTS spectrum of the sample was analyzed during the reaction under dark and light conditions (**Supplementary Figure 13)**.

Markedly, CO_2 molecules underwent recognizable adsorption and activation over the photocatalyst as indicated by the distinctive bicarbonate and carbonate peaks at 1030, 1335, 1400 1495 and 1515 cm^{-1} arising from CO_2 adsorption onto surface-active Mn-species of the Mn, C-ZnO CTSHSs sample under dark conditions (green stars in **Supplementary Figure 13**).[17,18] No detectable CO_2 reduction products were observed without light irradiation, suggesting that CO_2 is indeed photocatalytically reduced to valuable product. Upon light illumination, distinctive formaldehyde (HCO^-) and formate ($HCOO^-$) peaks at 1403, 1530, 2880 and 2980 cm^{-1} (red stars in **Supplementary Figure 13**), respectively[14,19,20], emerged on the DRIFTS spectra as intermediate products.

Moreover, characteristic peaks for CO are appeared within the chart at 2250 cm^{-1} (black star).[17,19] A sharp peak positioned at 1640 cm^{-1} was emerged after light switching. We attribute this peak to the O-H bending vibration of H_2O molecules susceptible for water oxidation by photogenerated holes at the valence band of ZnO.

Based on these findings, the photocatalytic CO_2 reduction paradigm can be drawn. Firstly, reliable adsorption of CO_2 molecules onto Mn^{n+} species is attained, followed by successful activation steps that culminate in the active radical anion intermediates *i.e.* $CO_2\cdot^-$ formation (see original manuscript, Figure 6c). Furthermore, this active intermediate will take a proton from water to yield HCOO· or ·COOH, according to its binding mode to the photocatalyst, which

could experience subsequent reduction steps by the photoexcited electrons from the photocatalyst resulting in HCOOH, and CO, respectively (**Supplementary Equations 2-8**):[21]

$$CO_2\,(ads) + Mn^{n+}\,(S) \rightarrow CO_2\cdot^{-}\,(ads) + Mn^{(n+1)+}\,(S) \qquad (2)$$

$$CO_2\cdot^{-}\,(ads) + H^{+}\,(ads) \rightarrow HCOO\cdot\,(ads) \qquad (3)$$

$$HCOO\cdot\,(ads) + e^{-} \rightarrow HCOO^{-}\,(ads) \qquad (4)$$

$$HCOO^{-}\,(ads) + H^{+}\,(ads) \rightarrow HCOOH\ \ (ads) \qquad (5)$$

$$HCOOH\ \ (ads) \rightarrow CO\ (ads) + H_2O\ (ads) \qquad (6)$$

$$Mn^{(n+1)+}\,(S) + e^{-} \rightarrow Mn^{n+}\,(S) \qquad (7)$$

At the other side, the water oxidation reaction could be expressed by equation 8:

$$2H_2O\ (ads) + 4h^{+} \rightarrow 4H^{+}\,(ads) + O_2\,(ads) \qquad (8)$$

The regeneration of the Mn^{n+} species is very critical to maintain sustainable and stable activity observed for the Mn-doped ZnO photocatalyst. Moreover, the charge trapping action manifested to restore the active Mn^{n+} species is of great essence for charge carrier's separation.

Supplementary Note 4

Fitting of TRPL. *: τ(average) represents the average lifetime for charge carriers generated on the photocatalyst upon light illumination, and such value is calculated based on the following equation[22] via substitution with the fitting parameters deduced from the TRPL results:

$$\tau(\mathrm{aver}age) = \frac{\mathrm{B}_1\tau_1^2 + \mathrm{B}_2\tau_2^2}{\mathrm{B}_1\tau_1 + \mathrm{B}_2\tau_2} \qquad (9)$$

where τ_1 and τ_2 are the lifetimes of radiative and non-radiative actions that occurred after the excitation process, respectively, and B_1 and B_2 are the pre-exponential factors.

Supplementary References

1. Shannon, R. D. & Prewitt, C. T. Effective ionic radii in oxides and fluorides. *Acta Crystallogr. Sect. B* **25**, 925–946 (1969).
2. Li, W. Advances in CO_2 Conversion and Utilization. 5–55 (American Chemical Society, 2010). doi:10.1021/bk-2010-1056.ch005.
3. Sharma, D. & Jha, R. Analysis of structural, optical and magnetic properties of Fe/Co co-doped ZnO nanocrystals. *Ceram. Int.* **43**, 8488–8496 (2017).
4. Wang, T. *et al.* A Co_3O_4 -embedded porous ZnO rhombic dodecahedron prepared using zeolitic imidazolate frameworks as precursors for CO_2 photoreduction. *Nanoscale* **8**, 6712–6720 (2016).
5. Etacheri, V., Roshan, R. & Kumar, V. Mg-doped ZnO nanoparticles for efficient sunlight-driven photocatalysis. *ACS Appl. Mater. Interfaces* **4**, 2717–2725 (2012).
6. Guo, Y. *et al.* Solution-based doping of manganese into colloidal ZnO nanorods. *J. Phys. Chem. C* **112**, 8832–8838 (2008).
7. Ajrina, N. *et al.* Mn-doping-induced photocatalytic activity enhancement of ZnO nanorods prepared on glass substrates. *Appl. Surf. Sci.* **439**, 285–297 (2018).
8. Sambandam, B., Michael, R. J. V. & Manoharan, P. T. Oxygen vacancies and intense luminescence in manganese loaded Zno microflowers for visible light water splitting. *Nanoscale* **7**, 13935–13942 (2015).
9. Wang, Y. *et al.* Synergistic effect of N-decorated and Mn^{2+} doped ZnO nanofibers with enhanced photocatalytic activity. *Sci. Rep.* **6**, 32711 (2016).
10. Gallegos, M. V., Peluso, M. A., Thomas, H., Damonte, L. C. & Sambeth, J. E. Structural and optical properties of ZnO and manganese-doped ZnO. *J. Alloys Compd.* **689**, 416–424 (2016).
11. Santos, V. P. *et al.* Structural and chemical disorder of cryptomelane promoted by alkali doping: Influence on catalytic properties. *J. Catal.* **293**, 165–174 (2012).
12. Liu, D. *et al.* Defect-related photoluminescence and photocatalytic properties of porous ZnO nanosheets. *J. Mater. Chem. A* **2**, 15377 (2014).
13. Wang, S. *et al.* C-doped ZnO ball-in-ball hollow microspheres for efficient photocatalytic and photoelectrochemical applications. *J. Hazard. Mater.* **331**, 235–245 (2017).
14. Low, J., Zhang, L., Tong, T., Shen, B. & Yu, J. TiO_2/MXene Ti_3C_2 composite with excellent photocatalytic CO_2 reduction activity. *J. Catal.* **361**, 255–266 (2018).
15. Aylmer-Kelly, A. W. B., Bewick, A., Cantrill, P. R. & Tuxford, A. M. Studies of electrochemically generated reaction intermediates using modulated specular reflectance spectroscopy. *Faraday Discuss. Chem. Soc.* **56**, 96–107 (1973).
16. Zhu, D. D., Liu, J. L. & Qiao, S. Z. Recent Advances in Inorganic Heterogeneous Electrocatalysts for Reduction of Carbon Dioxide. *Adv. Mater.* **28**, 3423–3452 (2016).
17. Xia, P., Zhu, B., Yu, J., Cao, S. & Jaroniec, M. Ultra-thin nanosheet assemblies of graphitic carbon nitride for enhanced photocatalytic CO_2reduction. *J. Mater. Chem. A* **5**, 3230–3238 (2017).
18. Fujita, S. I., Usui, M., Ito, H. & Takezawa, N. Mechanisms ofmethanol synthesis from carbon dioxide and from carbon monoxide at atmospheric pressure over Cu/ZnO. *J. Catal.* **157**, 403–413 (1995).
19. Chen, S., Yu, J. & Zhang, J. Enhanced photocatalytic CO_2 reduction activity of MOF-derived ZnO/NiO porous hollow spheres. *J. CO_2 Util.* **24**, 548–554 (2018).
20. Li, Z. *et al.* Highly Selective Conversion of Carbon Dioxide to Aromatics over Tandem Catalysts. *Joule* **3**, 570–583 (2019).
21. Yu, J., Xiang, Q. & Zhou, M. Preparation, characterization and visible-light-driven photocatalytic activity of Fe-doped titania nanorods and first-principles study for electronic structures. *Appl. Catal. B Environ.* **90**, 595–602 (2009).
22. Xia, P., Liu, M., Cheng, B., Yu, J. & Zhang, L. Dopamine Modified g-C_3N_4 and Its Enhanced Visible-Light Photocatalytic H_2-Production Activity. *ACS Sustain. Chem. Eng.* **6**, 8945–8953 (2018).
